# Deep Learning-Based Prediction of Individual Cell *α*-Dispersion Capacitance from Morphological Features

**DOI:** 10.3390/bios15110753

**Published:** 2025-11-10

**Authors:** Tae Young Kang, Soojung Kim, Yoon-Hwae Hwang, Kyujung Kim

**Affiliations:** 1 Institute for Future Earth, Pusan National University (PNU), Busan 46241, Republic of Korea; ty.kang@pusan.ac.kr; 2 Department of Cogno-Mechatronics Engineering, Pusan National University (PNU), Busan 46241, Republic of Korea; kimsoo9640@pusan.ac.kr; 3 Department of Nano Energy Engineering, Pusan National University (PNU), Busan 46241, Republic of Korea; yhwang@pusan.ac.kr; 4 Department of Optics and Mechatronics Engineering, Pusan National University (PNU), Busan 46241, Republic of Korea

**Keywords:** deep learning, cell morphology, single cell, α-dispersion capacitance, non-invasive cell characterization

## Abstract

The biophysical characteristics of cellular membranes, particularly their electrical properties in the α-dispersion frequency domain, offer valuable insights into cellular states and are increasingly important for cancer diagnostics through epidermal growth factor receptor (EGFR) expression analysis. However, a critical limitation in these electrical measurements is the confounding effect of morphological changes that inevitably occur during prolonged observation periods. These shape alterations significantly impact measured capacitance values, potentially masking true biological responses to epidermal growth factor (EGF) stimulation that are essential for cancer detection. In this study, we attempted to address this fundamental challenge by developing a deep learning method that establishes a direct computational relationship between cellular morphology and electrical properties. We combined optical trapping technology and capacitance measurements to generate a comprehensive dataset of HeLa cells under two different experimental conditions: (i) DPBS treatment and (ii) EGF stimulation. Our convolutional neural network (CNN) architecture accurately predicts 401-point capacitance spectra (0.1–2 kHz) from binary morphological images at low frequencies (0.1–0.8 kHz, < 10% error rate). This capability allows for the identification and subtraction of morphology-dependent components from measured capacitance changes, effectively isolating true biological responses from morphological artefacts. The model demonstrates remarkable prediction performance across diverse cell morphologies in both experimental conditions, validating the robust relationship between cellular shape and electrical characteristics. Our method significantly improves the precision and reliability of EGFR-based cancer diagnostics by providing a computational framework for a morphology-induced measurement error correction.

## 1. Introduction

The biophysical characteristics of cellular membranes, particularly their electrical properties, are fundamental determinants of numerous physiological processes such as ionic homeostatic regulation [1,2,3], signal transduction [4,5], nutrient transport [6], and cell volume control [7,8]. Notably, membrane capacitance measurements in the α-dispersion frequency domain allow precise characterisation of cellular states; that is, this frequency regime manifests the ionic environment and charge distribution dynamics proximal to cellular membranes [9,10,11,12]. These electrophysiological parameters have been extensively investigated to elucidate various cellular phenomena, including membrane protein conformational dynamics [13,14,15], receptor-mediated endocytosis [16,17], and cell cycle progression [18,19].

The measurement of cellular electrical properties has emerged as a promising method for cancer diagnostics, particularly through the analysis of epidermal growth factor receptor (EGFR) expression, which is frequently overexpressed in various malignancies [20,21,22]. Single-cell capacitance measurements following epidermal growth factor (EGF) stimulation can effectively differentiate between normal and cancerous cells based on their distinct electrical responses over time. However, a critical limitation of this diagnostic method is the morphological changes that occur over prolonged measurement periods. External electrical stimulation, alterations in the ionic environment, and cellular responses to EGF binding collectively induce significant morphological transformations that substantially impact the measured capacitance values, potentially compromising diagnostic accuracy.

Conventional population-based analyses of cellular electrical properties frequently yield averaged measurements that mask significant biological variation. The inherent heterogeneity of cellular populations, manifested as diverse cell states, sizes, and metabolic conditions, fundamentally limits the accuracy and biological relevance of aggregate measurements [23,24]. This recognition of cellular heterogeneity, which is particularly prominent in cancer cell populations where individual cells exhibit distinct characteristics and behaviours, highlights the critical importance of single-cell analysis in understanding fundamental cellular processes. Consequently, many sophisticated techniques have emerged for measuring electrical properties at the single-cell level. These include patch-clamp methodologies for precise membrane potential measurements [25,26], microfluidic platforms for controlled cellular manipulation [27,28], flow cytometry-based approaches for rapid single-cell analysis [29,30,31], and planar electrode substrates for label-free cell monitoring [32,33]. Among these methods, planar electrode substrates offer distinctive advantages for cellular analysis, allowing the real-time monitoring of adherent cells in their native state without requiring labelling or cellular manipulation. However, these advanced single-cell measurement techniques remain susceptible to errors arising from morphological variations during the measurement period. The critical issue is that the initial cellular morphology at the time of EGF administration frequently differs significantly from that at the end of the measurement protocol, introducing a significant source of error in cancer diagnostic applications that rely on precise capacitance measurements.

Recent advancements in artificial intelligence (AI) have resulted in the development of novel methodological frameworks for analysing biological systems. These computational approaches have successfully extracted meaningful patterns from complex biological data, including cell type classification [34,35,36], protein localisation [37,38,39], and cellular interaction networks [40,41,42]. The application of AI to address the morphology-dependent variations in cellular electrical measurements represents an untapped opportunity for improving diagnostic precision. A computational method capable of accurately predicting capacitance values based solely on initial morphological features would significantly improve the reliability of EGFR-based cancer diagnostics by eliminating the confounding effects of time-dependent morphological changes.

In this study, we present a novel method that combines non-invasive capacitance measurements with deep learning analysis to predict cellular capacitance based on morphological features. Our method focuses on the α-dispersion frequency regime, where cellular electrical properties correlate with membrane characteristics and ionic distributions. The developed convolutional neural network (CNN) architecture processes cell morphological images to predict the capacitance spectra of individual adherent cells while maintaining their viability during the measurement process. By establishing a direct computational relationship between the cellular morphology and electrical properties, our methodology can provide a method to identify and subtract morphology-dependent components from the measured capacitance changes during EGF stimulation.

## 2. Materials and Methods

### 2.1. Cell Culture and Treatments

HeLa cells obtained from the Korean Cell Line Bank (SNU, Seoul, Republic of Korea) were cultured on electrodes pretreated with 0.1% poly-L-lysine for 10 min and then sterilised with 70% ethanol and UV exposure. Cells were maintained in Dulbecco’s modified Eagle medium (Gibco™, 10569010, New York, NY, USA) supplemented with 10% foetal bovine serum (Gibco™, 16000036, New York, NY, USA) and 0.1% gentamicin (Gibco™, 15750060, New York, NY, USA) at 37 °C, 5% CO_2_, and 95% humidity

### 2.2. Electrode Fabrication

The substrate for measuring cell capacitance was fabricated by patterning a quartz surface using photolithography (LIT-2000, Nextron, Busan, Republic of Korea). A photoresist (AZ-GXR-601, AZ Electronic Materials, Luxembourg, Luxemburg) was applied to the quartz surface by spinning at 1000 rpm for 1 min, followed by hardening on a hot plate at 95 °C for 4 min. The coated layer was exposed to 352 nm ultraviolet light through a printed mask for 120 s, resulting in a linear electrode structure with a 50 μm gap length and width. Following a 30 s development in AZ 300 MIF and rinsing with deionised water, a thermal evaporation system was used to coat the substrate with Cr (20 nm) and Au (200 nm) layers. Deposition rates were maintained at 0.4 $\overset{˚}{A}$/s for Cr and 1.0–1.2 $\overset{˚}{A}$/s for Au under 10-6 mbar chamber pressure. Following metal deposition, the substrate underwent a lift-off process by immersing in acetone for 10 min with gentle agitation, removing excess photoresist and unwanted metal layers while preserving the patterned electrode structure. The completed electrodes were rinsed with isopropyl alcohol and deionised water to remove any residual acetone before drying with nitrogen gas.

### 2.3. Experimental Setup

**Figure 1:**
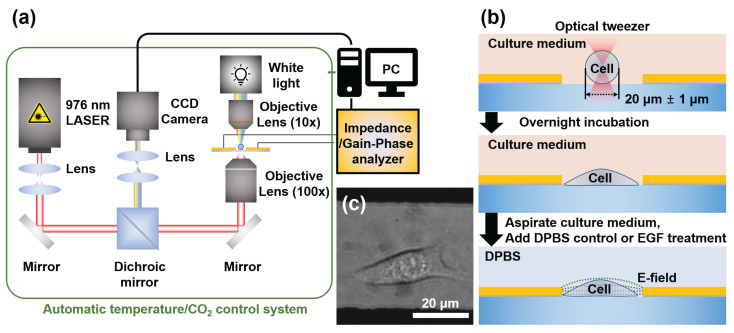
Experimental setup and cell-trapping process for single-cell capacitance measurements. (**a**) Schematic of the integrated optical tweezer system with capacitance sensing capability, featuring a 976 nm laser focused through a 100× objective and temperature/CO_2_ control. (**b**) Process diagram showing cell trapping, positioning between electrodes, and adhesion prior to measurement. (**c**) Representative image of HeLa cells successfully positioned and adhered within the electrode gap following the trapping procedure.

For cell capture and capacitance measurements, we developed an integrated system combining optical tweezers with automatic temperature/CO_2_ control. As shown in Figure 1a, the experimental setup featured an optical tweezer system that allowed precise single-cell manipulation and real-time electrical measurements. The substrate was positioned between an inverted 100× objective lens and a 10× condenser objective lens and aligned using a manually controlled XYZ stage with 1 μm positioning precision. A fibre-coupled 976 nm diode laser (BL976-PAG900, Thorlabs, Newton, MA, USA) focused through a 100× air microscope objective (MPLFLN 100× [0.9NA], Olympus, Tokyo, Japan) resulted in a trapping spot of approximately 1.8 μm. Laser power between 200 and 500 mW was used for cell manipulation in a controlled chamber environment (37 °C, 5% CO_2_).

Using this system, we successfully positioned individual HeLa cells at the geometric centre of the electrode gap with high precision (Figure 1c). This rigorous and standardized positioning protocol, a capability previously validated by our group for creating controlled multi-cell arrangements, is the key to eliminating positional variability as a confounding factor and ensuring the high repeatability of our measurements [43]. The optical trapping process typically requires less than one minute per cell, providing an efficient experimental throughput. Cell selection was performed using real-time charge-coupled device (CCD) real-time monitoring, which allowed us to identify and capture cells with well-defined smooth membranes as they gradually settled from suspension. To ensure consistency in our dataset, we specifically selected cells with diameters of approximately 20 ± 1 μm, avoiding excessively large or small cells that might indicate abnormal metabolic states or impending apoptosis. This strict size selection criterion served dual purposes: It ensured cellular health and provided dimensional consistency in our machine learning method. By controlling cell volume, we enabled our CNN model to infer three-dimensional cellular properties from two-dimensional binary images, improving our system’s predictive capabilities. After trapping, the cells were allowed to adhere to the poly-L-lysine coated surface for 30 min before being transferred to an external incubator for stable growth. Prior to the measurements, the culture medium was replaced with calcium- and magnesium-supplemented DPBS to minimise signal interference from serum components and prevent competition with EGF molecules in subsequent experiments (Figure 1b).

### 2.4. Capacitance Measurement

A single-cell capacitance was measured using an impedance/gain-phase analyser (HP4194A, Hewlett Packard, Palo Alto, CA, USA). Conductive copper tape (6 × 1 cm) established contact on each electrode end, with silver paste applied and hardened at 150 °C, 5% CO_2_ for 1 h, ensuring proper electrical connections. The measurement area was connected to the analyser using 16089D clip-lead connectors. Because the cell resistance exceeded 10 kΩ, data were collected as parallel capacitances ($C_{P}$). We focused on frequencies ranging from 0.1 to 2 kHz to capture membrane capacitance changes by leveraging α-dispersion properties [10,44]. To provide a clear physical basis for our measurements, it is essential to consider the standard equivalent circuit model for a cell at an electrode interface. As is well-established in this field, the system can be described by key components including the impedance of the electrical double-layer (Z_*elec*_) at the electrode-electrolyte interface, the resistance of the conductive medium (R_*m*_), and the cell’s own impedance (Z_*cell*_) [45]. A critical aspect of this model is that the total measured capacitance of the system is dominated by the very large electrical double-layer capacitance (C_*dl*_), which is typically orders of magnitude greater than the cell’s intrinsic membrane capacitance (C_*mem*_). Therefore, the primary parameter we report, |Δ$C_{P}$|, does not represent the cell’s membrane capacitance directly. Instead, it quantifies the cell-induced change in the total system capacitance relative to a baseline measurement taken with only DPBS. This differential value effectively isolates the electrical signature of the cell’s morphology and surface properties. The choice to collect data as parallel capacitance (C_*P*_) was a deliberate technical decision to optimize the signal-to-noise ratio (SNR) for our specific high-impedance system. For low-frequency measurements where the system’s parallel resistance (R_*P*_) is high, measuring in current mode (electrically equivalent to the parallel mode on our HP4194A analyzer) is fundamentally superior for minimizing the contribution of noises [46]. Each frequency sweep collected 401 data points (the maximum resolution supported by the analyser), with measurements completed in less than one minute to minimise stress on cells. Each measurement was performed in triplicate to ensure accuracy under the condition that the analyser automatically divided the frequency range into equal intervals for comprehensive spectral analysis. During the measurements, we used a low-voltage (100 mV) alternating electrical stimulation in the 0.1–2 kHz frequency range while maintaining physiologically relevant conditions (37 °C, 5% CO_2_) in a controlled chamber. This low-voltage, frequency-dependent method is essential for accurately probing the cellular dielectric properties within the α-dispersion regime, while ensuring the measurements remain in a linear response range and do not cause non-linear effects such as electroporation or undue cellular stress [47]. Although electrode polarisation significantly influences the absolute impedance at these low frequencies, its large and stable contribution is effectively removed by our use of a differential measurement (Δ$C_{P}$), which isolates the change caused solely by the cell. This non-invasive method allowed us to collect accurate electrical data while preserving the cells’ natural state.

### 2.5. Dataset Establishment

The dataset was constructed as follows: For each cell, we measured $C_{P}$ in the 0.1–2 kHz frequency range, obtaining 401 data points of |Δ$C_{P}$| and the baseline *C*_0_ measured from empty electrodes with DPBS stored in CSV format. Simultaneously, we captured images of each cell focused on a 50 × 50 μm area between the measuring electrodes. Cell images were preprocessed using a Gaussian filter to normalise them by removing noise, suspended particles in the medium, and artefacts generated by the optical system. Following noise reduction, we used a boundary detection algorithm with a threshold value of 0.5 to accurately identify cell perimeters [48]. Once the cell boundaries were determined, the interior regions were filled with black to produce distinct cell silhouettes against white backgrounds. These binary images were then standardised with a 64 × 64 pixels resolution while maintaining aspect ratios to optimise storage requirements and computational efficiency for subsequent analysis. A 90% dataset was used for training, with the remaining 10% dataset reserved for validating the model’s performance during training.

### 2.6. Training Model Development

**Figure 2:**
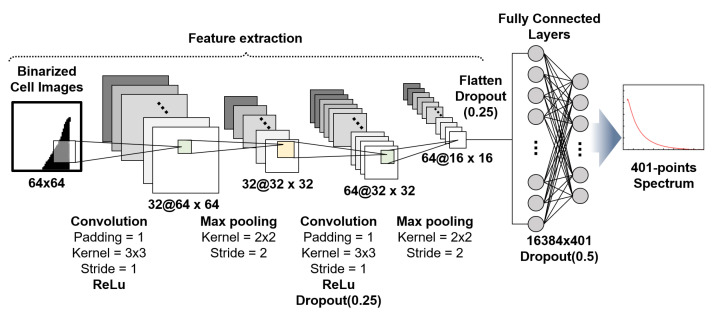
Convolutional neural network (CNN) architecture for predicting capacitance spectra from 64 × 64 binary cell images. The network uses two convolutional layers (each followed by max pooling) and one intermediate fully connected layer (16,384 neurons) to extract hierarchical features. These layers utilize the rectified linear unit (ReLU) activation function (symbolized as @). The final output layer consists of 401 neurons with a linear activation function to predict the continuous 401-point capacitance spectrum.

To investigate the relationship between cell morphology and electrical properties, we designed a CNN architecture to predict 401-point capacitance spectra from 64 × 64 binary cell images. As shown in Figure 2, our CNN model consisted of sequential convolutional and fully connected layers optimised for this prediction task. The network incorporated two convolutional layers for spatial feature extraction. The first layer uses 32 filters (3 × 3 size, stride 1, and padding 1) with rectified linear unit (ReLU) activation, followed by 2 × 2 max pooling. The second convolutional layer increased the depth to 64 filters with identical kernel parameters, followed by ReLU activation and similar pooling operations. These layers use nonlinear activations to capture hierarchical features from the input images before transitioning to fully connected layers. After flattening the convolutional output, the network included an intermediate fully connected layer with 16,384 neurones, followed by ReLU activation functions to maintain nonlinearity in these deeper representations. The final output layer comprised 401 neurones with linear activation (no ReLU), which corresponded directly to the target capacitance spectrum points across the frequency range. This linear output allows for unrestricted prediction of capacitance values, which is essential for accurately representing continuous spectral data. The model was implemented in PyTorch 2.5.1 and trained using the mean squared error (MSE) loss function with the Adam optimiser (learning rate: 0.001). We used a batch size of 32 for training with our dataset of 280 cells, and the training was conducted for 5000 epochs. To mitigate overfitting, strategic dropout regularisation was implemented throughout the network: 25% dropout following the second convolutional layer, 25% dropout after flattening, and 50% dropout before the final fully connected layer. This progressive increase in the dropout rate preserved essential feature information in the early layers while preventing co-adaptation in deeper representations.

## 3. Results and Discussion

**Figure 3:**
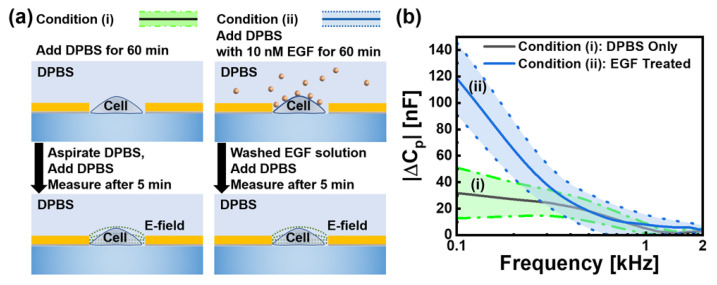
(**a**) Experimental protocols for two conditions: (i) Cells exposed only to DPBS and (ii) cells treated with 10 nM EGF followed by DPBS washing. In measurements, data were taken for 5 min after media exchange. (**b**) Frequency-dependent parallel capacitance change |Δ$C_{P}$| at frequencies from 0.1 to 2 kHz with the condition (i): DPBS control (n = 180, black/green) and the condition (ii): EGF-treated cells (n = 100, blue). At the frequency of 0.1 kHz, average values with conditions (i) and (ii) were 31 and 118 nF, respectively. Two shaded regions represent standard deviation, with baseline *C*_0_ measured from empty electrodes with DPBS.

Individual HeLa cells were precisely positioned within the electrode gap using our optical trapping system and subsequently maintained in culture conditions (37 °C, 5% CO_2_) for an hour to ensure stable attachment and physiological equilibrium prior to experimental measurements. Following successful cell positioning and culture, we conducted comprehensive electrical characterisation of 280 HeLa cells under two distinct experimental conditions, as shown in Figure 3a. We established two treatment protocols: condition (i) consisting of 180 cells treated solely with DPBS and condition (ii) comprising 100 cells exposed to 10 nM EGF for 60 min, followed by DPBS washing. Frequency-dependent parallel capacitance measurements showed significant differences between the two conditions. To provide the appropriate context for our results, we first clarify the physical origin of the measured capacitance values. The reported values in the nF range do not represent the intrinsic specific membrane capacitance of the cells, which would be in the pF range. Instead, the parameter we analyze, |Δ$C_{P}$|, is the magnitude of the change in the total system’s capacitance, measured relative to a baseline (*C*_0_) taken from empty electrodes with only DPBS. The large background capacitance of the system is dominated by the electrical double-layer (EDL) formed at the interface between the gold electrodes and the DPBS electrolyte. When a cell adheres to the electrode, it functions as an insulating particle that displaces the conductive medium and obstructs the current flow to that portion of the electrode surface. This shielding effect alters the properties of the dominant EDL, resulting in a measurable change in the total system capacitance. Therefore, the average value of approximately 31 nF at 0.1 kHz observed for cells under condition (i) (Figure 3b) represents the characteristic electrical signature of a standard HeLa cell’s physical presence—defined by its volume and morphology—within our specific measurement setup. In contrast, cells under condition (ii) showed significantly higher capacitance values, averaging 118 nF at the same frequency, a nearly 4-fold increase over the value under condition (i). This substantial capacitance enhancement can be attributed to the interaction of EGF with EGFRs on the cell surface. When EGF binds to these receptors, it causes conformational changes in membrane proteins, modifies ion channel activity, and alters the distribution of surface charges. These molecular events collectively alter the ionic distribution and charge density within the electrical double layer at the cell-medium interface, leading to the observed increase in the measured capacitance change |Δ$C_{P}$|.

Under both experimental conditions, we observed a consistent inverse relationship between capacitance and frequency, as shown by the declining curves in Figure 3b. This characteristic response follows established bioimpedance principles in the α-dispersion regime, where ionic displacement surrounding the cell membrane dominates the measured electrical properties. At these low frequencies, current preferentially flows around cells through the extracellular space, providing sensitive measurements of the electrical characteristics of the membrane and the surrounding ionic double layer. The α-dispersion phenomenon is caused by diffusion-controlled relaxation of ions adjacent to the charged cell membrane. When subjected to an alternating electric field, these ions underwent restricted movement and reorientation, resulting in the observed frequency-dependent response. Thus, measurements in this frequency range are particularly valuable for correlating cellular morphological characteristics to electrical response patterns. Because the cell membrane configuration, surface area, and topography directly influence the surrounding ionic environment, these structural features can be quantified through electrical measurements. All measurements were conducted under identical conditions, with consistent DPBS composition, temperature, and measurement timing (5 min after the final media exchange), ensuring that the observed capacitance differences were attributable specifically to the experimental treatments rather than environmental variables.

Following our electrical characterisation of HeLa cells, we developed a CNN capable of predicting the capacitance spectra from binary cell images. For our dataset of binary cell images paired with corresponding capacitance spectra, we used a 90/10 split ratio, allocating 90% of the data for training and reserving 10% for validation to monitor model performance during the training process. The training dataset included 162 cells for condition (i) and 90 cells for condition (ii), whereas the validation set contained 18 cells for condition (i) and 10 cells for condition (ii). We trained separate models for each experimental condition to account for the distinct electrical characteristics observed between DPBS- and EGF-treated cells.

**Figure 4:**
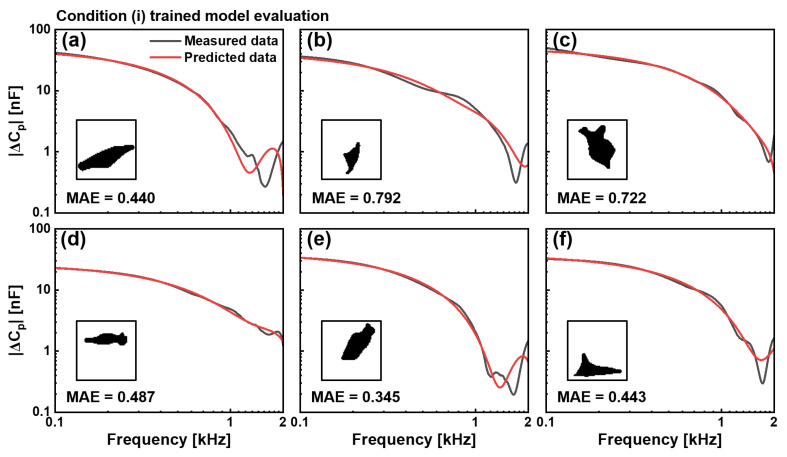
Evaluation of the CNN model trained with the condition (i), DPBS control cells. Comparison between measured (black) and predicted (red) capacitance spectra |Δ$C_{P}$| in the 0.1–2 kHz frequency range for six representative cells not included in the dataset. Binary cell images used as input for prediction are shown as insets. MAE values for six cells were (**a**) 0.440, (**b**) 0.792, (**c**) 0.722, (**d**) 0.487, (**e**) 0.345, and (**f**) 0.443. The model demonstrates consistent predictive capability across varied cell morphologies.

After the training process was completed, model performance was evaluated using the validation dataset. The resulting average MSE values were 0.426 for the condition (i) and 1.004 for the condition (ii). The higher error value observed in the EGF-treated cell model reflects the increased magnitude and variability of capacitance values in this experimental group, representing a more challenging prediction task. These MSE values were significantly higher than those typically observed in many deep learning applications [49], reflecting the challenges unique to predicting spectral responses in cellular systems. To analyse the practical utility of these trained models despite their high error metrics, we conducted a comprehensive evaluation using novel cells that were completely excluded from both the training and validation processes. To quantitatively assess prediction accuracy across the frequency spectrum, we used the mean absolute error (MAE), defined as follows:

(1) $$MAE=\frac{1}{N}\sum_{i=1}^{N}|y_{i}-{\hat{y}}_{i}|$$

where *N* represents the number of frequency points in each spectrum, *y*_*i*_ denotes the measured capacitance at the frequency point *i*, and ${\hat{y}}_{i}$ represents the corresponding predicted value. For the condition (i), DPBS-treated cells, our trained model demonstrated excellent predictive capabilities across diverse morphological presentations, and the evaluation of the trained model with the condition (i) was shown in Figure 4. Six representative test cells, which were completely excluded from the training dataset, were used to evaluate generalisation performance. The binary silhouettes of these cells, shown as insets, reveal substantial variations in their shape, size, and orientation. Comparing the measured capacitance spectra (black) with model predictions (red) in the 0.1–2 kHz frequency range showed significant correspondence in magnitude and spectral characteristics. The elongated cellular structure in test cell (a) yields an MAE value of 0.440, whereas the compact triangular morphology in test cell (b) results in an MAE value of 0.792. The irregular formation with protrusions in test (c) produced an MAE value of 0.722. In addition, morphological variants in the test cells (d)–(f) generated MAE of 0.487, 0.345, and 0.443, respectively.

**Figure 5:**
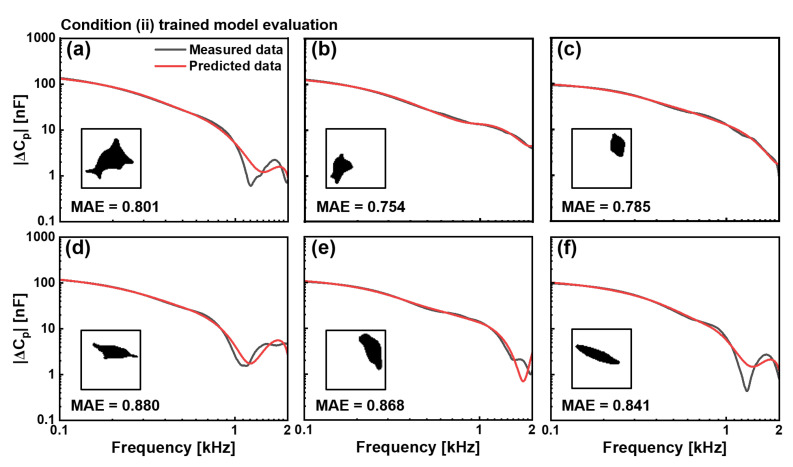
Evaluation of the CNN model trained with the condition (ii), EGF-treated cells. Comparison between measured (black) and predicted (red) capacitance spectra |Δ$C_{P}$| in the 0.1–2 kHz frequency range for six representative cells, not included in the training dataset. Binary cell images used as input for prediction are shown as insets. MAE values for six cells were (**a**) 0.801, (**b**) 0.754, (**c**) 0.785, (**d**) 0.880, (**e**) 0.868, and (**f**) 0.841. The model effectively predicts higher capacitance values characteristic of EGF-treated cells despite diverse morphologies.

For condition (ii), in EGF-treated cells, our model successfully addressed the considerably more challenging task of predicting substantially altered electrical properties, as shown in Figure 5. These six test cells exhibited significantly higher capacitance values across the frequency spectrum, indicating membrane modifications caused by EGF treatment. Despite its increased complexity, the model accurately captured both the magnitude- and frequency-dependent behaviours of capacitance spectra across morphologically diverse test cells. The irregular extended structure in test cell (a) produced an MAE value of 0.801, whereas the compact structure in test cell (b) resulted in an MAE value of 0.754. The smaller rounded profile in test cell (c) yields an MAE value of 0.785, and the elongated configurations in test cells (d), (e), and (f) generate MAE values of 0.880, 0.868, and 0.841, respectively.

Despite the relatively high MAE values observed during training, both models exhibited a consistent pattern of prediction accuracy that varied with frequency. Figure 4 and Figure 5 show that predictions demonstrated remarkable concordance with measured values at low frequencies (0.1–0.5 kHz), where cellular electrical properties are dominated by membrane characteristics and ionic distributions in the α-dispersion regime. This agreement progressively decreased as the frequency increased to 2 kHz, at which point the predictions showed greater instability and deviation from the measured values.

**Figure 6:**
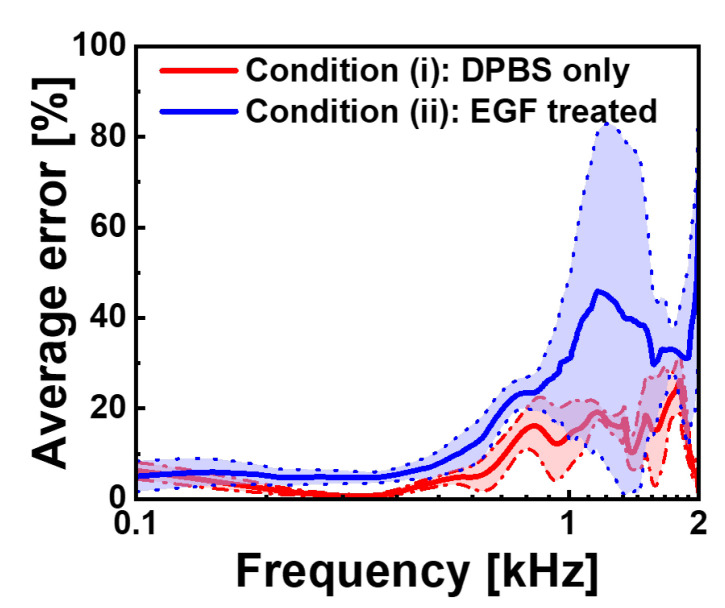
Frequency-dependent average prediction error rates for the CNN models. Average error percentages in the 0.1–2 kHz frequency range are shown for condition (i) (red) and condition (ii) (blue), with standard deviation boundaries represented by dotted lines. The error is calculated as the percentage difference between measured and predicted capacitance values. Both models demonstrate excellent performance at low frequencies (below 0.5 kHz) with error rates under 10%, while at high frequencies they show increased prediction uncertainty, particularly for EGF-treated cells, which reach approximately 45% error near 1.5 kHz compared to approximately 25% for control cells.

To quantitatively assess our models’ frequency-dependent prediction accuracy, we conducted an error analysis using 20 additional cells that were entirely excluded from both the training and validation datasets. The results are shown in Figure 6. The error percentage is computed as follows:

(2) $$Error\left[\%\right]=\frac{\left|Measured-Predicted\right|}{Measured}\times 100\left[\%\right]$$

For the DPBS-treated cells (blue), the average error remained consistently below 10% in the frequency range of 0.1–0.8 kHz, indicating exceptional prediction accuracy in this region. As the frequency increased beyond 0.8 kHz, error rates gradually increased, reaching approximately 25% at 1.5 kHz before exhibiting greater fluctuations near 2 kHz. EGF-treated cells (red) showed a similar trend but with higher overall error magnitudes, starting with errors below 10% at the lowest frequency and progressively increasing to approximately 45% at 1.5 kHz. The shaded regions representing the standard deviations around these average error curves show the variability across the 20 test cells, with EGF-treated cells displaying wider error bands, consistent with their greater electrical heterogeneity.

This frequency-dependent error profile revealed a fundamental relationship between cellular morphology and electrical properties. The superior prediction accuracy at low frequencies indicates that two-dimensional morphological features captured in binary images effectively encode membrane-related electrical characteristics that predominate in the α-dispersion regime. As frequency increases, prediction accuracy decreases, indicating a growing influence of intracellular structures and complex membrane organisational factors that cannot be fully represented in two-dimensional silhouettes. Despite overall high MSE values observed during training, our models maintain substantial predictive power for cellular electrical characteristics, which are relevant to membrane-associated phenomena, as evidenced by a consistent pattern of relatively low errors in the 0.1–0.8 kHz frequency range across both experimental conditions.

A notable aspect of our methodology is the dimensional consistency maintained in our dataset through strict cell-size selection criteria. Although our CNN model operated solely on two-dimensional binary silhouettes, the controlled initial volume constraints appeared to allow for the implicit inference of three-dimensional properties during training. This suggests that, under standardised experimental conditions, planar morphological features provide enough information for computational estimation of volumetric cellular characteristics that influence electrical properties. The practical implementation of our correction methodology involves a straightforward arithmetic process; that is, by capturing cell images at both the beginning and end of the measurement protocols, researchers can predict capacitance values attributable solely to the morphology at each time point. The difference between these predicted values represents the morphology-dependent component, which can be subtracted from the measured capacitance changes to isolate the true biological response to EGF stimulation, improving diagnostic precision.

## 4. Conclusions

In this study, we showed the feasibility of predicting single-cell capacitance spectra from binary morphological images using CNNs. Our method may resolve the critical limitation of EGFR-based cancer diagnostics, that is, the confounding effects of morphological changes that occur during prolonged capacitance measurements. By combining the optical trapping technology with capacitance measurements, we established a comprehensive dataset of HeLa cells under two distinct experimental conditions: DPBS treatment and EGF stimulation. This method enabled us to investigate the relationship between cellular morphology and electrical properties while maintaining precise control over the measurement conditions. The central achievement of our methodology lies in its ability to accurately predict the electrical characteristics of cells based solely on their morphological states. This capability fundamentally transforms the reliability of single-cell electrical measurements for cancer diagnosis by allowing for correction of measurement errors caused by time-dependent morphological variations. A potential consideration of our deep learning approach is the size of the training dataset, which consists of 280 high-fidelity single-cell measurements. While modest compared to large-scale computer vision tasks, our methodology aligns with the principles of a data-centric AI approach, where the emphasis shifts from massive data quantity to high data quality, particularly in specialized domains where data acquisition is inherently limited. Indeed, developing robust models from datasets in the range of 10^2^ to 10^3^ relevant data points is an accepted practice in such fields [50]. Our protocol, which prioritizes the consistency of each data point through meticulous optical tweezing, is a direct implementation of this data-centric principle. Demonstrating strong generalization performance, our CNN architecture accurately predicted 401 points of capacitance spectra across frequencies from 0.1 to 2 kHz using only binary morphological images. The model proved particularly effective in the critical low-frequency range (0.1–0.8 kHz), maintaining an error rate below 10%. This capability allows for the identification and subtraction of morphology-dependent components from measured capacitance changes, effectively isolating true biological responses from morphological artefacts. By providing a computational framework for correcting morphology-induced measurement errors, our method improves the precision and reliability of EGFR-based cancer diagnostics, potentially contributing to improved cancer detection and characterisation methodologies.

## Data Availability

The raw data supporting the conclusions of this article will be made available by the authors on request.

## Abbreviations

The following abbreviations are used in this manuscript:

AI	Artificial intelligence
CCD	Charge-coupled device
CNN	Convolutional neural network
DPBS	Dulbecco’s phosphate buffered saline
EGF	Epidermal growth factor
EGFR	Epidermal growth factor receptor
MAE	Mean absolute error
MSE	Mean squared error
